# Supplementary material for: Murine Falcor/LL35 lncRNA Contributes to Glucose and Lipid Metabolism In Vitro and In Vivo
Source: Biomedicines. 2022 Jun 13;10(6):1397. doi: 10.3390/biomedicines10061397 (PMC9220108; doi:10.3390/biomedicines10061397)
Supplement: Supplementary file 1 [file biomedicines-10-01397-s001.zip › Supplementary_Materials.pdf]

# Murine Falcor/LL35 lncRNA contributes in glucose and lipid metabolism *in vitro* and *in vivo*

**Evgeniya Shcherbinina [1], Tatiana Abakumova [1], Daniil Bobrovskiy [2], Ilia Kurochkin [1], Ksenia Deinichenko [3], Elena Stekolshchikova [1], Nickolay Anikanov [1], Rustam Ziganshin [4], Pavel Melnikov [5], Ekaterina Khrameeva [1], Maria Logacheva[1], Timofei Zatsepin [1, 6], and Olga Sergeeva [1,*]**

[1] Skolkovo Institute of Science and Technology, 121205 Moscow, Russia;
[2] Faculty of Bioengineering and Bioinformatics, Lomonosov Moscow State University, 119234 Moscow, Russia
[3] Institute of Biomedical Chemistry, 119121 Moscow, Russia
[4] Shemyakin-Ovchinnikov Institute of Bioorganic Chemistry, 117997 Moscow, Russia
[5] Serbsky National Medical Research Center for Psychiatry and Narcology, 119034 Moscow, Russia
[6] Department of Chemistry, Lomonosov Moscow State University, 119991 Moscow, Russia

* Correspondence: O.Sergeeva@skoltech.ru

## Supplementary Materials

**Supplementary Table S1**. List of ASOs for *in vitro* study.

| Name | Sequence |
|---|---|
| LL35 ASO 1 | ususgscscsAsTsCsAsTsGsTsTsGsTsascscsusg |
| LL35 ASO 2 | cscscscsusCsTsCsAsGsTsGsCsTsGsgsasascsc |
| LL35 ASO 3 | ususasgsgsTsGsGsCsAsGsTsTsCsAsgsgsasgsa |
| LL35 ASO 4 | gsuscsgsgsTsAsTsCsAsGsTsTsGsCsasgsasgsa |
| LL35 ASO 5 | usgsususgsusAsCsCsTsGsGsCsCsAsGsTscsasgscsusgsc |
| Control Luc ASO | tscsgsasasgsTsAsCsTsCsAsGscsgstsasasg |

Capital letters indicate 2’-deoxynucleotides, lowercase letters indicate 2’-O-methylribonucleotides, s – phosphorothioate groups.

**Supplementary Table S2**. List of ASOs for *in vivo* study.

| Name | Sequence |
|---|---|
| LL35 ASO 1 | oUsoUsoGsoCsoCsAsTsCsAsTsGsTsTsGsTsoAsoCsoCsoUsoG3galnac |
| LL35 ASO 2 | oCsoCsoCsoCsoUsCsTsCsAsGsTsGsCsTsGsoGsoAsoAsoCsoC3galnac |
| LL35 ASO 3 | oUsoUsoAsoGsoGsTsGsGsCsAsGsTsTsCsAsoGsoGsoAsoGsoA3galnac |
| LL35 ASO 4 | oGsoUsoCsoGsoGsTsAsTsCsAsGsTsTsGsCsoAsoGsoAsoGsoA3galnac |
| LL35 ASO 5 | oAsoCsoCsoUsoGsoGsCsCsAsGsTsCsAsGsCsTsoGsoCsoUsoGsoA3galnac |
| Control Luc ASO | oUsoCsoGsoAsoAsoGsTsAsCsTsCsAsGsoCsoGsoUsoAsoAsoG3galnac |

Capital letters indicate 2’-deoxynucleotides, o – 2′-O-methoxyethyl (MOE), s – phosphorothioate groups, 3galnac – triple *N*-acetylgalactosamine (GalNAc) TEG.

**Supplementary Table S3**. List of PCR primers used in the study.

| Name | Forward, 5’→3’ | Reverse, 5’→3’ |
|---|---|---|
| GAPDH | TGCACCACCAACTGCTTAGC | GGATGCAGGGATGATG |
| ACTB | CGGTTCCGATGCCCTGAGGCTCTT | CGTCACACTTCATGATGGAATTGA |
| LL35 | CTCAGTGCTGGAACCCCATCTGG | CTCCAAGGCAACACAATGGGAC |
| HK2 | ATGATCGCCTGCTTATTCACG | CGCCTAGAAATCTCCAGAAGGG |
| LDHA | TGTCTCCAGCAAAGACTACTGT | GACTGTACTTGACAATGTTGGGA |
| ENO2 | AGGTGGATCTCTATACTGCCAAA | GTCCCCATCCCTTAGTTCCAG |
| PFKFB2 | ACATGCTCATGGGCTTCCTAT | TGAGGTAGCGTGTTAGTTTCT |
| PFKFB3 | CCCAGAGCCGGGTACAGAA | GGGGAGTTGGTCAGCTTCG |
| GLUT1 | CAGTTCGGCTATAACACTGGTG | GCCCCCGACAGAGAAGATG |
| GLUT2 | TCAGAAGACAAGATCACCGGA | GCTGGTGTGACTGTAAGTGGG |
| PDK1 | AGGATCAGAAACCGGCACAAT | GTGCTGGTTGAGTAGCATTCTAA |
| G6PC | CTGTTTGGACAACGCCCGTAT | AGGTGACAGGGAACTGCTTTA |
| PEPCK | CTGCATAACGGTCTGGACTTC | CAGCAACTGCCCGTACTCC |
| PGC1α | TATGGAGTGACATAGAGTGTGCT | CCACTTCAATCCACCCAGAAAG |
| SIRT1 | CATCAGCTATCGTTCGTCCAG | CCACAGCGTCATATCATCCAG |
| MYC | ATGCCCCTCAACGTGAACTTC | CGCAACATAGGATGGAGAGCA |
| EGR1 | TCGGCTCCTTTCCTCACTCA | CTCATAGGGTTGTTCGCTCGG |
| FOXJ1 | CCCTGACGACGTGGACTATG | GCCGACAGAGTGATCTTGGT |
| NOTCH1 | CCGTGTAAGAATGCTGGAACG | AGCGACAGATGTATGAAGACTCA |
| NOTCH4 | CTCTTGCCACTCAATTTCCCT | TTGCAGAGTTGGGTATCCCTG |
| HEY1 | CCGACGAGACCGAATCAATAAC | TCAGGTGATCCACAGTCATCTG |
| HES1 | CCAGCCAGTGTCAACACGA | AATGCCGGGAGCTATCTTTCT |
| IKBA | TGGCCAGTGTAGCAGTCTTG | GACACGTGTGGCCATTGTAG |
| CDH1 | CAGGTCTCCTCATGGCTTTGC | CTTCCGAAAAGAAGGCTGTCC |
| ABCA9 | TCGATAGATGCAGTGAGAGTCA | CACAAGGAGCTGAATGGTCTTT |
| MYOCBP1 | GATCGCTACCGGAGGATTTACA | CAATGCCGCACGTTACAGTA |
| AGXT | AAGGCATCCAGTATGTGTTCCA | TTCCGGTTAGAAAGGAGTCCC |
| CDC7 | AACAGCGTGATGAGGGAAACT | CGCTCTGAATCCTGGTGTG |
| CENPI | AGGGTTACTAGAAACTCCCAGC | GCGTGTAGAATCTTCCACTGAA |
| CHEK1 | TTCCACCAACTCATGGCAGG | GCGTTCACGATTATTATGCCGAA |
| GADD45G | GGGAAAGCACTGCACGAACT | AGCACGCAAAAGGTCACATTG |
| GGT6 | TCCTCAATCAACAGTTCCTTTGG | CAAGGTGCAAGTCATGGCTTT |
| GSTM2 | ACACCCGCATACAGTTGGC | TGCTTGCCCAGAAACTCAGAG |
| GTSE1 | TTTTGGGCCTGTTGGACATAAA | CTCAAGGTGCAAGGGCTACC |

| | | |
|---|---|---|
| IL33 | ATTTCCCCGGCAAAGTTCAG | AACGGAGTCTCATGCAGTAGA |
| MDM2 | TAAAGTCCGTTGGAGCGCAAA | CTGCTGCTTCTCGTCATATAACC |
| PDE4D | AACACTGCACTCCTGTAATGAAG | TGCTTGTTCCAACTGTCTGAAG |
| PFKM | TGTGGTCCGAGTTGGTATCTT | GCACTTCCAATCACTGTGCC |
| PINK1 | GGCTTCCGTCTGGAGGATTAT | AACCTGCCGAGATATTCCACA |
| PSMC6 | CCTGTCAGAACAGATTCGGGA | GCAAACAGCCTTTTGGAGGTATT |
| RHOH | CTACAAACCCACGGTGTACGA | CCGGATACTTCTGAAGGCGT |
| ZFP36 | CCGAATCCCTCGGAGGACTT | GAGCCAAAGGTGCAAAACCA |
| PCYT1B | AGACACTGTCGATACGTGGAT | TCACGGACAATTCTGGTGATG |
| CHPT1 | CAAACTCTTGCTCTCCCTTAGG | CTGAGACGTAAGTCTGCCAATG |
| CEPT1 | ATGAGTGGGCATCGGTCAAC | GTGGTGTCGGTAACTGAAACAA |
| PISD | CATACTGCTCCTGTCCGATCC | TTCCGTTCCCTGTACTTCTCATA |
| ETNK1 | CTGTTCACAGATGGGATCACAA | CGCGGAAACTTTTCACTTCCTC |
| ETNK2 | CGGTGGAACAGGACGACATC | AGGCCAATAGCTTGTTGGTGA |
| LPCAT1 | AGAGCAGAGACATCCCAATCT | CCAGGTTTGAAGGTAATGAGGC |
| LPCAT3 | CTACCCGTTGGCTCTGTTTTAC | TGAAGCACGACACATAGCAAG |
| PLA2G4A | CAGCACATTATAGTGGAACACCA | AGTGTCCAGCATATCGCCAAA |
| SMPD1 | TGGGACTCCTTTGGATGGG | CGGCGCTATGGCACTGAAT |
| ASAH1 | TCCGTGGCACACCATAAATCT | TCCACTTGGCACAAATGTATTCA |

Capital letters indicate 2’-deoxynucleotides.

**Supplementary Table S4**. List of primary antibodies used in the study.

| Antibody | Ref # | Manufacturer | Dilution |
|---|---|---|---|
| β-actin | MA1-140 | Thermo Fisher Scientific MA, Waltham, USA | 1:5000 |
| GAPDH | 2118 | Cell Signaling, Danvers, MA, USA | 1:3000 |
| IκBα | 9242 | Cell Signaling, Danvers, MA, USA | 1:1000 |
| p105/p50 (NF-κB) | 13586 | Cell Signaling, Danvers, MA, USA | 1:1000 |
| AKT1 | 2938 | Cell Signaling, Danvers, MA, USA | 1:1500 |
| pAKT1 | 4060 | Cell Signaling, Danvers, MA, USA | 1:1500 |
| Jagged1 | 2620T | Cell Signaling, Danvers, MA, USA | 1:1000 |
| Pck1/PEPCK | ab70358 | Abcam, Cambridge, UK | 1:1000 |

**Supplementary Table S5**. Gene expression table for LL35 KD versus Luc control in AML12 cells and Luc control versus LL35 KD in mouse liver. Provided as separate file.

**Supplementary Table S6**. Polar metabolite and lipid abundances for AML12 cells and mouse liver after LL35 depletion. Provided as separate file.

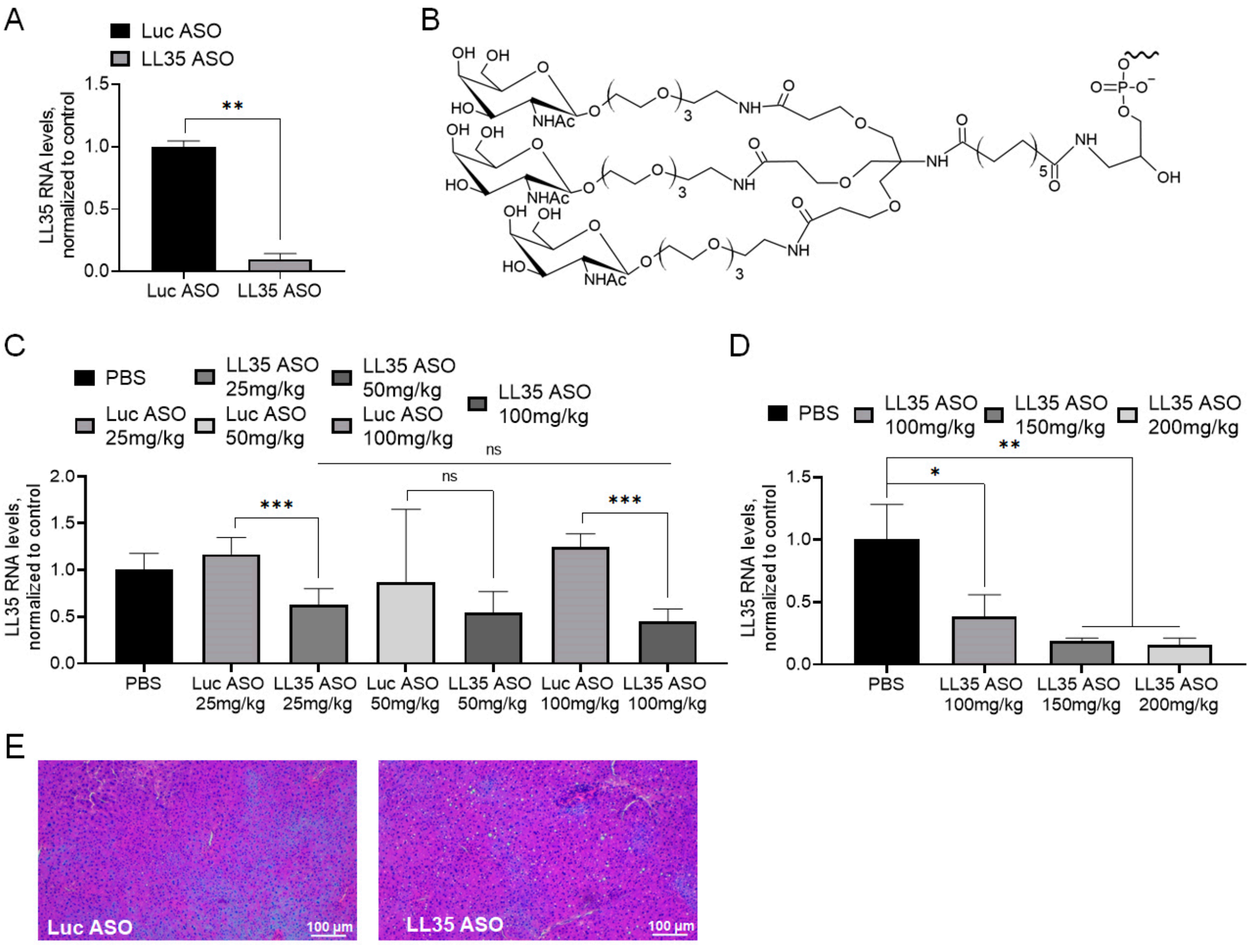


**Supplementary Figure S1**. LL35 knockdown *in vitro* and *in vivo.* **A.** LL35 knockdown on the 2nd day after AML12 cells treatment with 20nM mix of 5 ASOs specific to LL35 (LL35 ASO), Luc ASO – control. **B.** Schematic structure of GalNAc-ASO conjugates used for LL35 depletion in vivo. **C.** LL35 knockdown on the 2nd day of mice injection with different concentrations of GalNAc-ASO conjugates (LL35 ASO), Luc ASO, PBS - control. **D**. LL35 knockdown on the 5th day of mice injection with different concentrations of GalNAc-ASO conjugates (LL35 ASO), PBS - control. Normalization on ACTB gene. **E.** Hematoxylin-eosin (H&E) staining of liver samples after LL35 depletion (LL35 ASO). Luc ASO – control. Results show mean ± SD. n.s.—not significant. * $p < 0.05$, ** $p < 0.01$ and *** $p < 0.001$.

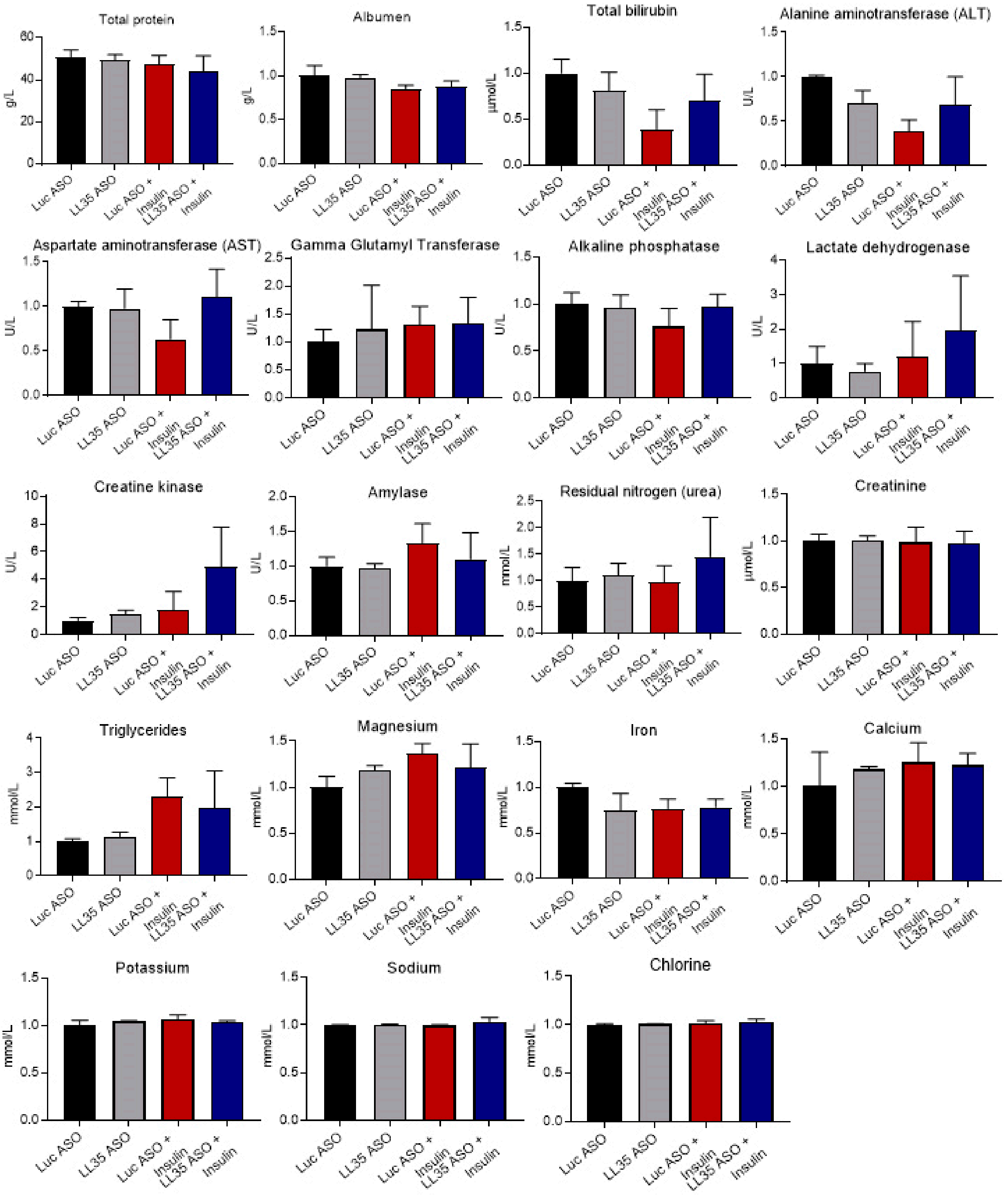


**Supplementary Figure S2**. Biochemical markers analyzed in animal blood on the 5th day after LL35 depletion by injection of GalNAc-ASO conjugates (LL35 ASO), Luc ASO - control. Luc ASO + Insulin and LL35 ASO + Insulin indicates groups of animals which were treated with 1U/kg insulin 1 hour before blood sampling for analysis.

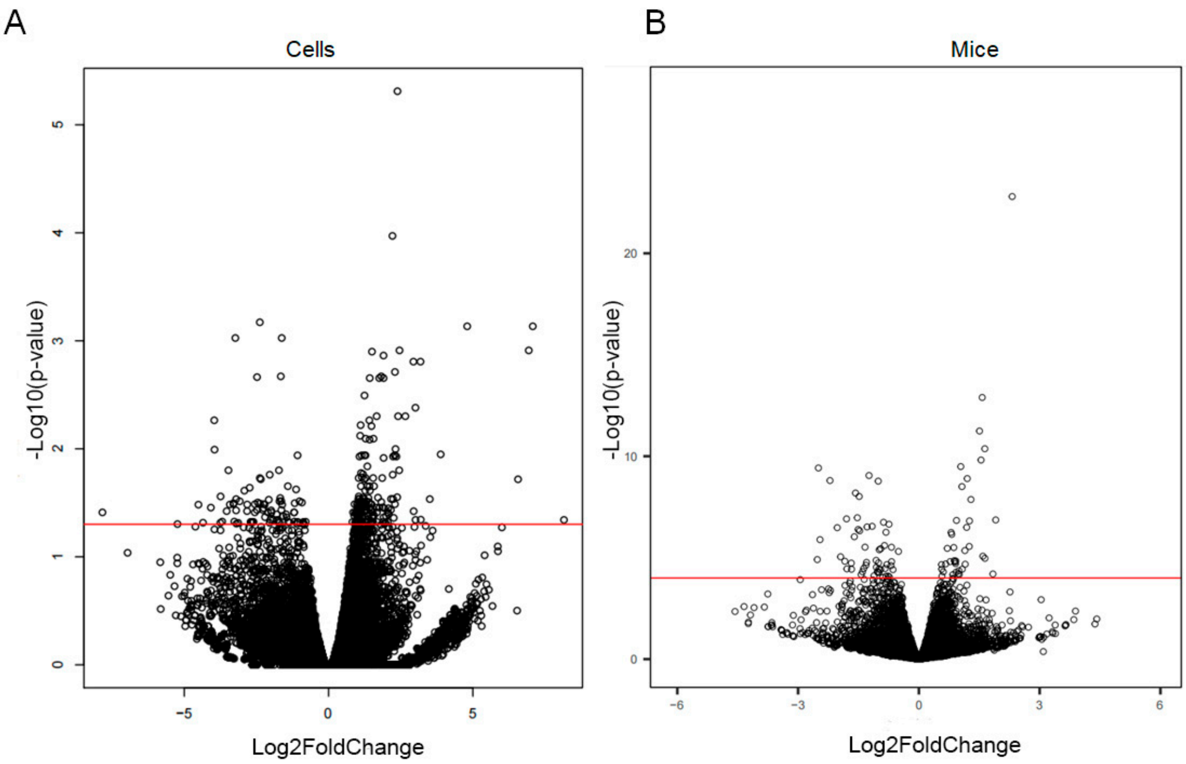


**Supplementary Figure S3.** Volcano plots of gene expression after LL35 depletion by ASOs mix in **A**. AML12 cells, day 2; **B**. mouse liver, day 5.

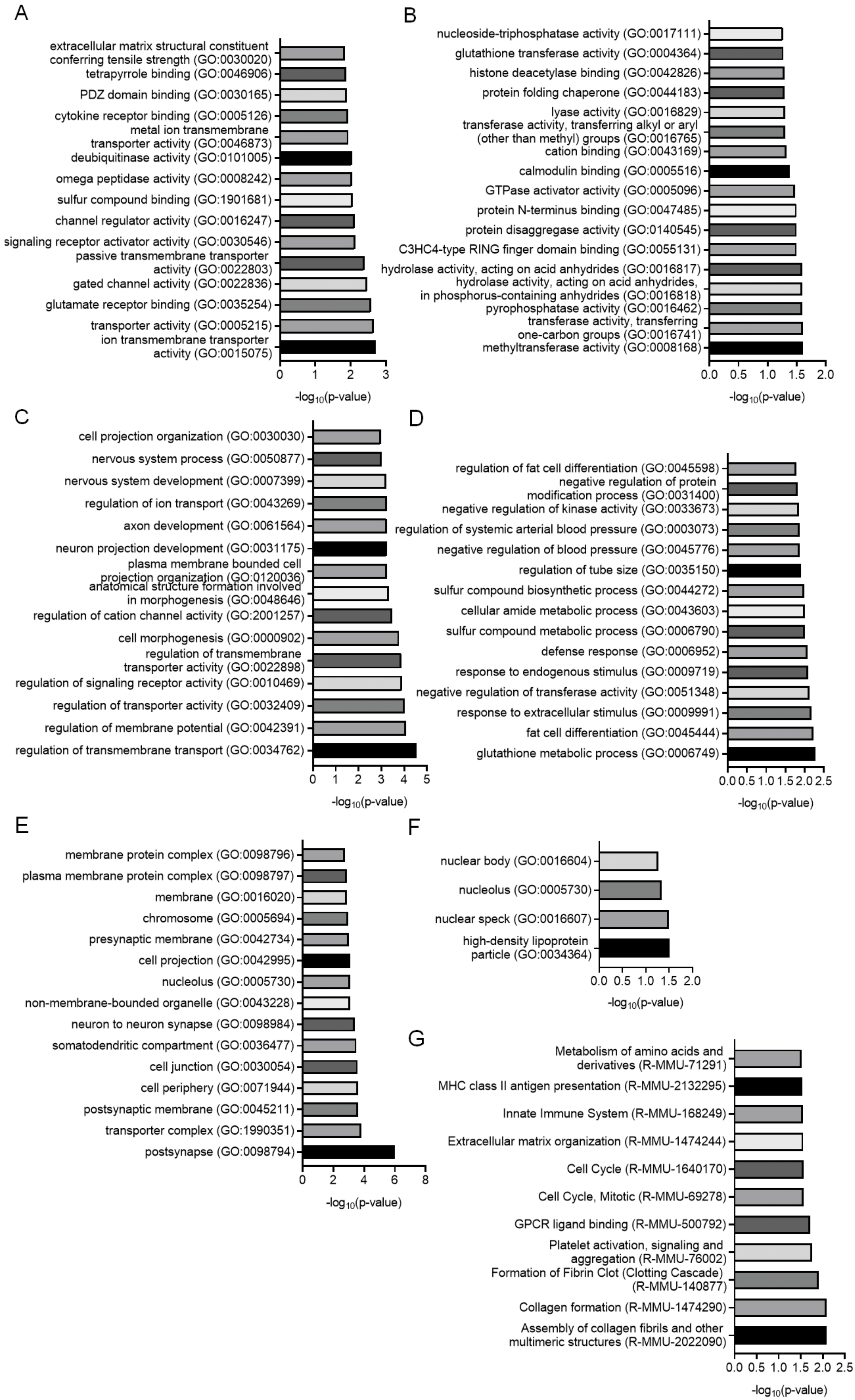

A
extracellular matrix structural constituent conferring tensile strength (GO:0030020)
tetrapyrrole binding (GO:0046906)
PDZ domain binding (GO:0030165)
cytokine receptor binding (GO:0005126)
metal ion transmembrane transporter activity (GO:0046873)
deubiquitinase activity (GO:0101005)
omega peptidase activity (GO:0008242)
sulfur compound binding (GO:1901681)
channel regulator activity (GO:0016247)
signaling receptor activator activity (GO:0030546)
passive transmembrane transporter activity (GO:0022803)
gated channel activity (GO:0022836)
glutamate receptor binding (GO:0035254)
transporter activity (GO:0005215)
ion transmembrane transporter activity (GO:0015075)
0 1 2 3
-log10(p-value)
B
nucleoside-triphosphatase activity (GO:0017111)
glutathione transferase activity (GO:0004364)
histone deacetylase binding (GO:0042826)
protein folding chaperone (GO:0044183)
lyase activity (GO:0016829)
transferase activity, transferring alkyl or aryl (other than methyl) groups (GO:0016765)
cation binding (GO:0043169)
calmodulin binding (GO:0005516)
GTPase activator activity (GO:0005096)
protein N-terminus binding (GO:0047485)
protein disaggregase activity (GO:0140545)
C3HC4-type RING finger domain binding (GO:0055131)
hydrolase activity, acting on acid anhydrides (GO:0016817)
hydrolase activity, acting on acid anhydrides, in phosphorus-containing anhydrides (GO:0016818)
pyrophosphatase activity (GO:0016462)
transferase activity, transferring one-carbon groups (GO:0016741)
methyltransferase activity (GO:0008168)
0.0 0.5 1.0 1.5 2.0
-log10(p-value)
C
cell projection organization (GO:0030030)
nervous system process (GO:0050877)
nervous system development (GO:0007399)
regulation of ion transport (GO:0043269)
axon development (GO:0061564)
neuron projection development (GO:0031175)
plasma membrane bounded cell projection organization (GO:0120036)
anatomical structure formation involved in morphogenesis (GO:0048646)
regulation of cation channel activity (GO:2001257)
cell morphogenesis (GO:0000902)
regulation of transmembrane transporter activity (GO:0022898)
regulation of signaling receptor activity (GO:0010469)
regulation of transporter activity (GO:0032409)
regulation of membrane potential (GO:0042391)
regulation of transmembrane transport (GO:0034762)
0 1 2 3 4 5
-log10(p-value)
D
regulation of fat cell differentiation (GO:0045598)
negative regulation of protein modification process (GO:0031400)
negative regulation of kinase activity (GO:0033673)
regulation of systemic arterial blood pressure (GO:0003073)
negative regulation of blood pressure (GO:0045776)
regulation of tube size (GO:0035150)
sulfur compound biosynthetic process (GO:0044272)
cellular amide metabolic process (GO:0043603)
sulfur compound metabolic process (GO:0006790)
defense response (GO:0006952)
response to endogenous stimulus (GO:0009719)
negative regulation of transferase activity (GO:0051348)
response to extracellular stimulus (GO:0009991)
fat cell differentiation (GO:0045444)
glutathione metabolic process (GO:0006749)
0.0 0.5 1.0 1.5 2.0 2.5
-log10(p-value)
E
membrane protein complex (GO:0098796)
plasma membrane protein complex (GO:0098797)
membrane (GO:0016020)
chromosome (GO:0005694)
presynaptic membrane (GO:0042734)
cell projection (GO:0042995)
nucleolus (GO:0005730)
non-membrane-bounded organelle (GO:0043228)
neuron to neuron synapse (GO:0098984)
somatodendritic compartment (GO:0036477)
cell junction (GO:0030054)
cell periphery (GO:0071944)
postsynaptic membrane (GO:0045211)
transporter complex (GO:1990351)
postsynapse (GO:0098794)
0 2 4 6 8
-log10(p-value)
F
nuclear body (GO:0016604)
nucleolus (GO:0005730)
nuclear speck (GO:0016607)
high-density lipoprotein particle (GO:0034364)
0.0 0.5 1.0 1.5 2.0
-log10(p-value)
G
Metabolism of amino acids and derivatives (R-MMU-71291)
MHC class II antigen presentation (R-MMU-2132295)
Innate Immune System (R-MMU-168249)
Extracellular matrix organization (R-MMU-1474244)
Cell Cycle (R-MMU-1640170)
Cell Cycle, Mitotic (R-MMU-69278)
GPCR ligand binding (R-MMU-500792)
Platelet activation, signaling and aggregation (R-MMU-76002)
Formation of Fibrin Clot (Clotting Cascade) (R-MMU-140877)
Collagen formation (R-MMU-1474290)
Assembly of collagen fibrils and other multimeric structures (R-MMU-2022090)
0.0 0.5 1.0 1.5 2.0 2.5
-log10(p-value)

**Supplementary Figure S4.** Gene ontology analysis (p-value<0.05) of transcriptome data after LL35 knockdown: A. molecular functions MF, AML12 cells; B. MF, murine liver; C. cellular components (CC), AML12 cells; D. CC, murine liver; E. biological pathways (BP), AML12 cells; F. BP, murine liver. G. Top representative pathways obtained by PANTHER Reactome analysis of differentially expressed genes ($|log_2FC|>0.8$, adjusted p-value <0.1) in lung epithelium isolated from null LL35 mice [23].

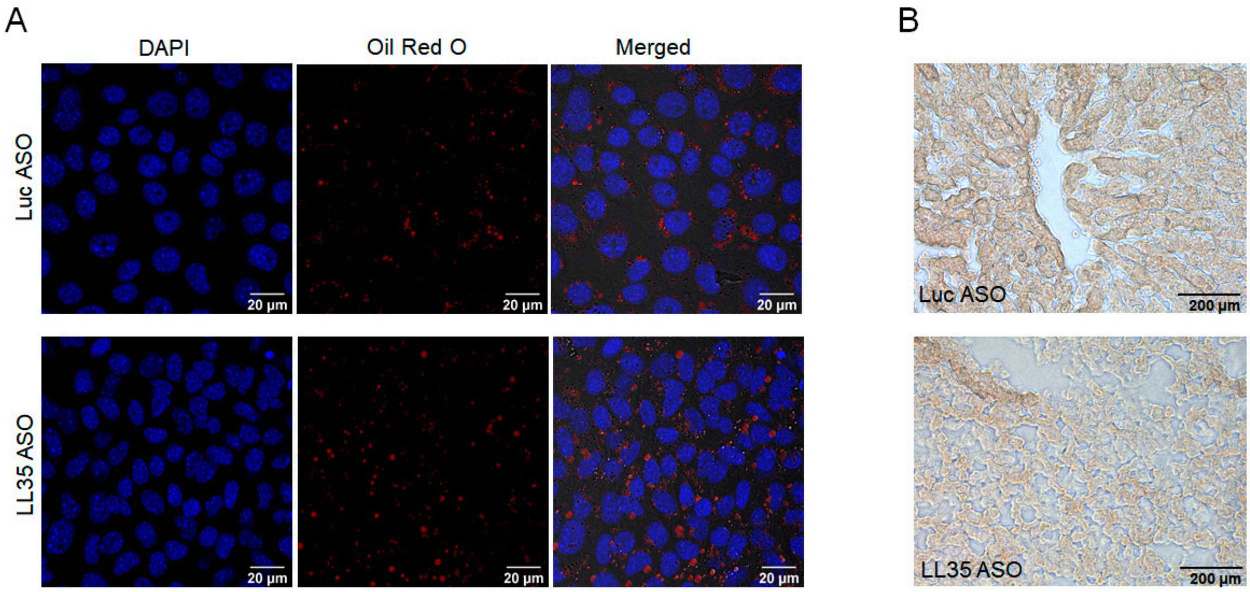


**Supplementary Figure S5.** Lipid droplets staining with Oil Red O after LL35 depletion. (LL35 ASO) in **A.** AML12 cells (nucleus were stained with DAPI); **B.** in mouse liver. Control – Luc ASO.

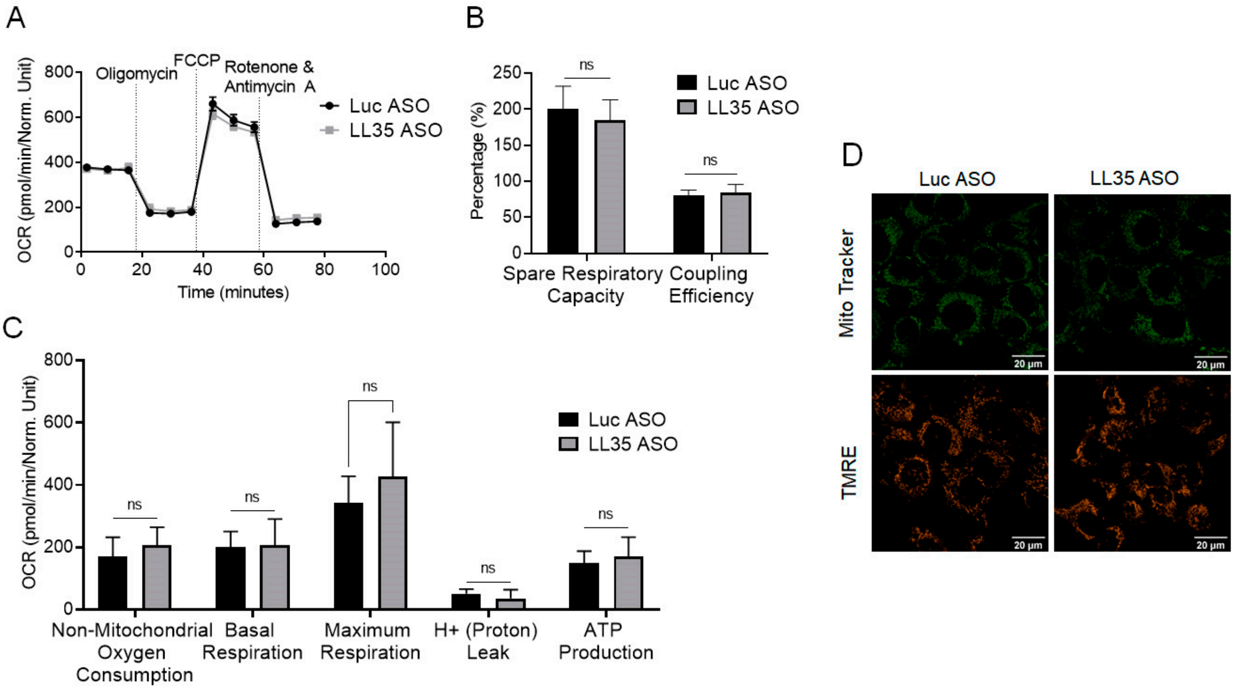


**Supplementary Figure S6.** Characterization of mitochondria function after LL35 depletion *in vitro*. **A.** The average time curve for OCR after subsequent injections of 1µM oligomycin, 1µM FCCP and 1µM rotenone/antimycin A. Each data point represents an OCR value used for calculations. **B.** Individual parameters for cell respiration in %, including spare respiratory

capacity and coupling efficiency for AML12 cells after LL35 depletion (LL35 ASO) and control (Luc ASO). **C.** Individual parameters for cell respiration, including non-mitochondrial oxygen consumption, basal respiration, maximum respiration, proton leak, ATP production for AML12 cells after LL35 depletion (LL35 ASO) and control (Luc ASO). **D.** AML12 cells mitochondria staining with TMRE and Mito Tracker green after LL35 depletion and in control. Results show mean ± SD. n.s.—not significant.

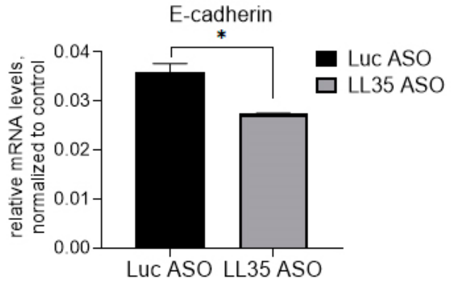


**Supplementary Figure S7.** E-cadherin mRNA expression levels after LL35 depletion in comparison with luciferase control measured by RT-qPCR. Results show mean ± SD. * $p < 0.05$.

## Supplementary Methods

1. *Library preparation for RNA-seq analysis*

As a starting quantity for library preparation, we used four micrograms of total RNA previously extracted from AML12 cells and liver samples using TRIzol reagent (Thermo Fisher Scientific, Waltham, MA, USA), followed by precipitation with isopropanol, according to the manufacturer's protocol. First, we performed rRNA depletion using a NEBNext rRNA Depletion Kit (NEB E6310L, New England Biolabs, Ips-wich, MA, USA) according to the manufacturer’s protocol with the following changes. Before the depletion we fragmented the total RNA to ~200 nt-long RNA fragments by incubation at 95 °C for 5.5 min in 100 mM Tris (pH 8.0) and 2 mM $MgCl_2$. After the fragmentation RNA was purified by 96 % ethanol precipitation with a 1/10 volume of 3 M sodium acetate. One microgram of fragmented and purified RNA was used for rRNA depletion reaction by NEBNext rRNA Depletion Kit (NEB E6310L, New England Biolabs, Ips-wich, MA, USA). The second purification of RNA after rRNA depletion step was again performed by 96 % ethanol precipitation with a 1/10 volume of 3 M sodium acetate (instead of purification using magnetic beads as described in manufacturer’s instructions). Then we measured RNA concentration using a NanoDrop™ OneC Spectrophotometer (Thermo Fisher Scientific, Waltham, MA, USA), and 300 ng of purified RNA were used for the sequencing library preparation with a NEBNext Ultra II Directional RNA Library Prep Kit for Illumina (NEB 7760, New England Biolabs, Ipswich, MA, USA), according to the manufacturer’s protocol. The synthesized double-stranded cDNA was purified with AMPure XP magnetic beads (A63881, Beckman Coulter, Brea, CA, USA). Library quality was evaluated on a Bioanalyzer2100 (Agilent Technologies, Santa Clara, CA, USA). Libraries were sequenced using a HiSeq4000 (Illumina, San Diego, USA) instrument in 5 nt single-read mode. Data processing and analysis are described in the main text.

2. *LC-MS-based proteomics*

Protocol for reduction, alkylation and digestion of the proteins in solution were optimized from Kulak et *al.* [29] with minor modifications. Briefly, sodium deoxycholate (SDC) lysis, reduction and alkylation buffer 100 mM Tris-HCl, 1 % (w/v) SDC, 10 mM TCEP and 20 mM 2-chloroacetamide (pH 8.5) were added to a cell sample. First, we heated each sample for 10 min at 95 °C, second, we added in 1:100 (w/w) ratio the equal volume of trypsin solution in 100 mM Tris-HCl (pH 8.5) and samples have been subjected to overnight digestion at 37 °C. Then 1 % trifluoroacetic acid (TFA) was used for peptides acidification for SDB-RPS binding, and 20 µg of sample was loaded on three 14-gauge StageTip plugs. After adding the equal volume of ethyl acetate the StageTips were centrifuged at 400 g. Then the StageTips were washed with a 100 µl of 1 % TFA/ethyl acetate mixture and 100 µl of 0.2 % TFA, and peptides were eluted using 50 µl of the elution solution (50 % acetonitrile, 45 % water and 5 % ammonia). The collected material was vacuum-dried and stored at -80 °C.

Before analysis peptides were dissolved in 2 % acetonitrile/ 0.1 % TFA solution and sonicated for 2 min in ultrasonic water bath. Then samples were placed in a home-made trap column 20 × 0.1 mm with Inertsil ODS3 3 µm sorbent (GL Sciences, Tokyo, Japan), in the loading buffer contained 2 % ACN, 98 % $H_2O$, 0.1 % TFA at 10 µl/min flow and separated in a home-packed [30] fused-silica column 300 × 0.1 mm packed with Reprosil PUR C18AQ 1.9 (Dr. Maisch, Ammerbuch-Entringen, Germany) at RT into an emitter prepared with P2000 Laser Puller (Sutter, Atlanta, GA, USA). Ultimate 3000 Nano LC System (Thermo Fisher Scientific, Waltham, MA, USA) coupled to the Q Exactive Plus Orbitrap mass spectrometer (Thermo Fisher Scientific, Waltham, MA, USA) via a nanoelectrospray source (Thermo Fisher Scientific, Waltham, MA, USA) was used for reverse-phase chromatography. After loading the peptides in a loading solution contained 98 % 0.1 % (v/v) formic acid and 2 % (v/v) acetonitrile they were eluted with a linear gradient as follows: 3–35 % solution B (0.1 % (v/v) formic acid, 80 % (v/v) acetonitrile) for 105 min; 35-55 % B for 18 min, 55-99 % B for 0.1 min, 99 % B during 10 min, 99-2 %B for 0.1 min at a flow rate of 500 nl/min. The preequilibration of the column with solution A (0.1 % (v/v) formic acid, 2 % (v/v) acetonitrile) for 10 min was performed after each gradient. We used the following MS1 parameters: 70K resolution, 350-2000 scan range, 30 msec maximum injection time, AGC target – $3 \times 10^6$. For ions isolation 1.4 m/z window, preferred peptide match and isotope exclusion were used. Dynamic exclusion was set to 30 s. MS2 fragmentation was performed in HCD mode at 17.5K resolution with HCD collision energy 29 % and 80msec maximum injection time, AGC target – $1 \times 10^5$. Settings for charge exclusion: unassigned, 1, >7.

3. *Cell lines and liver tissue extraction protocol for lipids and metabolites analysis*

AML12 cells were plated into 150 mm cell culture dish (1 dish per biological replica, 3 replicas per condition). At day 2 after transfection with ASOs mix (20nM each) for LL35 or Luc using Lipofectamine RNAiMax (Invitrogen, Waltham, MA, USA) cells were collected and washed with PBS solution twice, supernatant was discarded and cell pellets were frozen at -80 °C until extraction. On extraction day cells were thawed on ice for several minutes and randomized. For liver samples 10-15 mg liver tissue pieces were dissected from the frozen tissue samples on dry ice, weighed, randomized and transferred to a cooled 2 ml Precellys tubes with 6 zirconium oxide beads (Bertin Technologies, Montigny-le-Bretonneux, France). Extraction blank samples were inserted in the end of main batch, consisting of an empty tube without cell or tissue sample. The extraction buffer (methanol/ methyl-*t*-butyl ether (MTBE) (1/3, v/v)) with spiked lipid standards (0.5 µg/ml): triacylglycerol TAG(15:0/18:1-d7/15:0) (791648C, Avanti Lipids, Birmingham, AL, USA), diacylglycerol DAG(15:0/18:1-d7) (791647C, Avanti Lipids, Birmingham, AL, USA), ceramide Cer(d18:1-d7/15:0) (860681, Avanti Lipids, Birmingham, AL, USA), lyso phosphocholine LPC(18:1-d7) (791643C, Avanti Lipids, Birmingham, AL, USA), phosphoglycerol PG(15:0/18:1-d7) (791640C, Avanti Lipids, Birmingham, AL, USA), phosphocholine PC(15:0/18:1-d7) (791637C, Avanti Lipids, Birmingham, AL, USA), phosphoethanolamine PE(15:0/18:1-d7) (791638C, Avanti Lipids, Birmingham, AL, USA) was

added. Additionally, for liver samples SM (791649, Avanti Lipids, Birmingham, AL, USA), PS (791639, Avanti Lipids, Birmingham, AL, USA), Chol (700041, Avanti Lipids, Birmingham, AL, USA) were added to extraction buffer. In case of cell culture samples after vigorous vortexing for 1 min, mixture was sonicated (35-kHz frequency) in ice-cold bath (Bandelin Sonorex Super RK 100 H, BANDELIN electronic GmbH & Co. KG) for 10 minutes, vortexed for 10 min and sonication repeated for 10 min. After addition of 0.5 ml of extraction buffer to liver tissue samples, homogenization of the tissue pieces was performed on a Precellys Evolution Tissue Homogenizer with dry ice-filled Cryolys cooling system (Bertin Technologies, Montigny-le-Bretonneux, France). Then another 0.5 ml of extraction buffer was added and homogenates were sonicated for 10 min in an ice-cooled sonication bath (Bandelin Sonorex Super RK 100 H, BANDELIN electronic GmbH & Co. KG) and incubated for 40 min at 4 °C on an orbital shaker, sonication was repeated for 10 min.

Phase separation was induced by adding 700 µL of water/methanol (3/1, v/v) mixture with the following standards (6.7 µg/ml): L-Glutamic acid-$^{13}$C5 (604860, Sigma-Aldrich, MO, USA), methionine-methyl-13C, d3 (299154, Sigma-Aldrich, MO, USA), GMP-$^{15}$N5 (662674, Sigma-Aldrich, MO, USA). Extract was vortexed for 15 min at 4 °C, then centrifuged for 10 min at 18000×g, at 4 °C. A total of 540 µL (for cells) or 300 µL (for liver) of the upper layer, containing most of the lipids, was collected to an Eppendorf tube. 900 µL (for cells) or 1000 µL (for liver) of polar lower phase was collected in a separate Eppendorf tube. Solvents were evaporated under reduced pressure at 30 °C in the Speed Vac concentrator (mode V-HV, Eppendorf Concentrator plus Complete System, Hamburg, Germany). Dried samples were stored in -80 °C until mass spectrometry analysis. Pellets were reconstituted in 200 µL of acetonitrile/isopropanol (7/3 (v/v)) or in 100 µL of 20 % acetonitrile in water for lipids and polar metabolites analysis, respectively. After brief rigorous vortexing, samples were shaken for 10 min at 4 °C, sonicated (35-kHz frequency) in an ice-cooled sonication bath for 10 min, and centrifuged 10 min at 18000×g at 4 °C. Extracts were diluted prior lipidome analysis as follows: 1:10 and 1:5 (1:50 and 1:3 for liver samples) with acetonitrile:isopropanol (7:3 (v:v)) for positive and negative mode, respectively. Extracts were diluted prior to metabolome analysis as follows: 1:10 and 1:3 (1:5 and 1:2 for liver samples) with 20 % acetonitrile in water for positive and negative mode, respectively. For quality control of samples, equal volumes of each diluted sample were additionally collected in a pool sample.

*4. Liquid chromatography/mass spectrometry analysis of lipidome*

Liquid chromatography/mass spectrometry system consisted of Waters Acquity I-Class UPLC system (Waters, Manchester, UK) and Q Exactive orbitrap mass spec-trometer (Thermo Fisher Scientific, Waltham, MA, USA) equipped with a heated electro-spray ioniza-tion (HESI) probe was used for lipids and metabolites data acquisition from AML12 cells and liver tissue samples. We did a lipid separation at 60 °C on a reverse phase ACQUITY UPLC BEH C8 Column (2.1×100 mm, 1.7 µm, Waters co., Milford, MA, USA) equipped with Vanguard pre-column at flow rate of 0.4 mL/min. The resulting mobile phases consisted of water with 10 mM ammonium acetate, 0.1 % formic acid (Buffer A), and a mixture of acetonitrile and isopropanol (7:3) with 10 mM ammonium acetate, 0.1 % formic acid (Buffer B). For negative polarity of detection acetic acid was used instead of formic acid as mobile phase additive in the same concentration. The gradient elution was used for separation with the following profile: 1 min 55 % B, 3 min linear gradient from 55 to 80 % B, 8 min linear gradient from 80 to 85 % B, and 3 min linear gradient from 85 to 100 % B. The column was repeatedly equilibrated with 55 % B for 4.5 min after washing for 4.5 min with 100 % B. The volume for injection – 3 µL. Mass spectra were acquired in positive mode with a mass range of m/z 120–1500 (positive mode) and 200-2300 (negative mode) at a mass resolving power of 140,000 Full Width Half Maximum (FWHM) (m/z 200). The mass spectrometer was operated with following parameters for full scan and data dependent acquisition (DDA) modes: ion transfer tube temperature, 320 °C; vaporizer temperature, 350 °C; spray voltage, 4.5 kV; S-lens RF level, 60; AGC target value,

5E5, sheath gas ($N_2$) flow rate, 45 arbitrary units (a.u.); auxiliary gas ($N_2$) flow rate, 20 a.u.; sweep gas ($N_2$) flow rate, 4 a.u. Data was acquired on the profile mode. External mass axis calibration (Pierce positive and negative calibration solutions, Thermo Fisher Scientific) without the use of the specific lock masses was employed. DDA Top-10-acquisition mode was used for pooled samples to access fragmentation patterns of lipids and confirm lipid classes. Operating parameters were established as follows: resolution 17500 at m/z 200, AGC: 2e4, IT: 100 ms, mass isolation window: 1.2 Da, retention time window width: expected time ± 1 minute, stepped normalized collision energy: 15, 20, 25 %, dynamic exclusion 12 s, inclusion: on, customize tolerances: 10 ppm. The spectra were recorded in the profile mode. The same procedure was repeated with inclusion lists to confirm putative lipid annotations.

*5. Liquid chromatography/mass spectrometry analysis of metabolome*

Liquid chromatography/mass spectrometry system consisted of Waters Acquity I-Class UPLC system (Waters, Manchester, UK) and Q Exactive orbitrap mass spec-trometer (Thermo Fisher Scientific, USA) equipped with a heated electro-spray ioniza-tion (HESI) probe was used for lipids and metabolites data acquisition from AML12 cells and liver tissue samples. We did a polar metabolites separation at 40 °C on a ZIC-HILIC Column (2.1×100 mm, 3.5 µm, SeQuant, Merck, Germany) equipped with pre-column at a flow rate of 0.4 mL/min. The resulting mobile phases consisted of water/acetonitrile mixture (95/5, v/v) with 20 mM ammonium acetate, 0.2 % acetic acid (Buffer A), and a mixture of acetonitrile/water (95/5, v/v) with 20 mM ammonium acetate, 0.2 % acetic acid (Buffer B). The gradient elution was used for separation with the following profile: 0-1.5 min 0 % B, 4.5 min linear gradient from 0 to 15 % B, 7 min linear gradient from 15 to 45 % B, and 5 min linear gradient from 45 to 100 % B. The column was repeatedly equilibrated with 0 % B for 5 min after washing for 2 min with 100 % B. The volume for injection – 3 µL. Mass spectra were acquired with a mass range of m/z 60–1000 at a mass resolving power of 140,000 Full Width Half Maximum (FWHM) (m/z 200) for both polarities. The mass spectrometer was operated with following parameters: ion transfer tube temperature, 320 °C; vaporizer temperature, 350 °C; spray voltage, 4.5 kV; S-lens RF level, 60; AGC target value, 5e5, sheath gas (N2) flow rate, 45 arbitrary units (a.u.); auxiliary gas (N2) flow rate, 20 a.u.; sweep gas (N2) flow rate, 4 a.u. Data was acquired on the profile mode. External mass axis calibration (Pierce positive and negative calibration solutions, Thermo Fisher Scientific) without the use of the specific lock masses was employed. DDA-acquisition Top-10 mode was used for pooled samples to access fragmentation patterns of polar metaboltites. Operating parameters were established as follows: resolution 17500 at m/z 200, AGC: 2e4, IT: 100 ms, mass isolation window: 1.2 Da, retention time window width: expected time ± 1 minute, stepped normalized collision energy: 15, 35, 55 %, dynamic exclusion 12 s, inclusion: on, customize tolerances: 10 ppm. The spectra were recorded in the profile mode.
